# Thermoregulation and survival during sepsis: insights from the cecal ligation and puncture experimental model

**DOI:** 10.1186/s40635-024-00687-8

**Published:** 2024-11-10

**Authors:** Luis H. A. Costa, Isis P. Trajano, Patricia Passaglia, Luiz G. S. Branco

**Affiliations:** 1 Department of Oral and Basic Biology, School of Dentistry of Ribeirão Preto - University of São Paulo, Avenida Bandeirantes, Ribeirão Preto, SP 14040-902 Brazil; 2 Department of Neurology, Beth Israel Deaconess Medical Center, Harvard Medical School, Boston, USA; 3 Department of Physiology, Medical School of Ribeirão Preto, University of São Paulo, Ribeirão Preto, SP Brazil

**Keywords:** Fever, Hypothermia, PGE2, Cytokines, Mortality, Hypothalamus

## Abstract

**Background:**

Sepsis remains a major global health concern due to its high prevalence and mortality. Changes in body temperature (Tb), such as hypothermia or fever, are diagnostic indicators and play a crucial role in the pathophysiology of sepsis. This study aims to characterize the thermoregulatory mechanisms during sepsis using the cecal ligation and puncture (CLP) model and explore how sepsis severity and ambient temperature (Ta) influence Tb regulation and mortality. Rats were subjected to mild or severe sepsis by CLP while housed at thermoneutral (28 °C) or subthermoneutral (22 °C) Ta, and their Tb was monitored for 12 h. Blood and hypothalamus were collected for cytokines and prostaglandin E_2_ (PGE_2_) analysis.

**Results:**

At 28 °C, febrile response magnitude correlated with sepsis severity and inflammatory response, with tail vasoconstriction as the primary heat retention mechanism. At 22 °C, Tb was maintained during mild sepsis but dropped during severe sepsis, linked to reduced UCP1 expression in brown adipose tissue and less effective vasoconstriction. Despite differences in thermoregulatory responses, both Ta conditions induced a persistent inflammatory response and increased hypothalamic PGE_2_ production. Notably, mortality in severe sepsis was significantly higher at 28 °C (80%) compared to 22 °C (0%).

**Conclusions:**

Our findings reveal that ambient temperature and the inflammatory burden critically influence thermoregulation and survival during early sepsis. These results emphasize the importance of considering environmental factors in preclinical sepsis studies. Although rodents in experimental settings are often adapted to cold environments, these conditions may not fully translate to human sepsis, where cold adaptation is rare. Thus, researchers should carefully consider these variables when designing experiments and interpreting translational implications.

**Graphical Abstract:**

**Figure:**
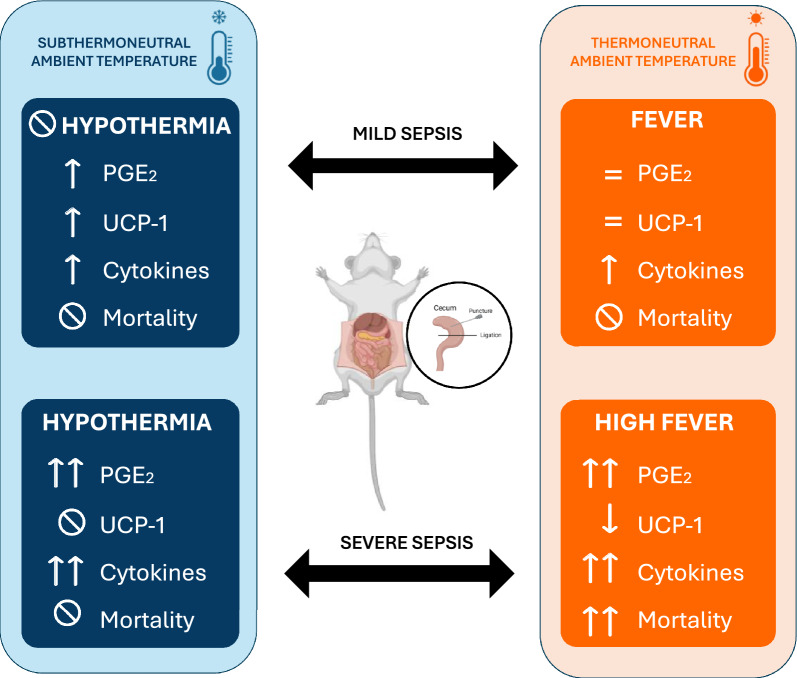
Graphical representation of the thermoregulatory and inflammatory responses to mild and severe sepsis in rats housed at thermoneutral (28 °C) and subthermoneutral (22 °C) ambient temperatures (Ta). The model highlights distinct body temperature outcomes: fever and hypothermia. At thermoneutral Ta, severe sepsis induces a high fever, associated with increased hypothalamic PGE2, cytokine levels, and mortality, while mild sepsis leads to a moderate fever. In contrast, at subthermoneutral Ta, severe sepsis results in hypothermia with decreased UCP1 expression and lower mortality, whereas mild sepsis maintains normal body temperature. The impact of Ta on sepsis severity, thermoregulatory mechanisms, and survival is emphasized.

## Background

Body temperature (Tb) control is a fundamental aspect of survival for animals. An intricate, and yet to be fully deciphered response orchestrated by the brain activates peripheral mechanisms that regulate heat loss and/or production in order to maintain core body temperature [1]. The hypothalamus, more specifically the preoptic area (POA), is a fundamental component within the hierarchical organization of the neuronal circuits regulating thermoeffector activity, integrating central and systemic information and, through other brain connections, controlling both autonomic and behavioral thermoregulatory responses [2–4]. Similar to other mammals, rodents have more than one thermoregulatory effector to keep body temperature within a narrow regulated range. Maintenance of Tb depends on the dynamic activation of thermoeffectors that result in heat dissipation, conservation or generation. As previously demonstrated [5], vasomotor changes in the tail contribute significantly to heat dissipation, with up to 20% of heat production lost by the tail in rats. Other areas that lack fur, such as the feet and distal portions of the limbs, also play an important role in regulating dry heat loss, given that it constitutes approximately 10% of the rat’s surface area [6]. Conversely, heat can be generated by brown adipose tissue (BAT) activation, a process known as non-shivering thermogenesis. This energy-consuming process is mediated by the uncoupling protein 1 (UCP1), which is highly expressed in BAT. UCP1 increases inner mitochondrial membrane conductance for protons and dissipates its gradient without generating ATP, converting the energy of substrate oxidation into heat [7]. BAT thermogenesis has been shown to be a key thermoeffector during adaptation to cold environments, but also plays a role in thermoregulation during other stimuli such as stress, exercise, and hibernation [8].

Alterations in body temperature are an important clinical manifestation, being considered a symptom of disease even by Hippocrates in the fifth–fourth century B.C. [9]. Both an increase (fever) and a decrease (hypothermia) in the normal range of Tb can be observed during an infectious condition. Sepsis, an exacerbated systemic inflammatory response following an infection, is considered a major health concern worldwide, particularly in intensive care units (ICUs), and even the first medical guidelines recognize Tb greater than 38 °C or less than 36 °C as a major diagnostic clue for this disease [10]. During sepsis, inflammatory mediators, like cytokines, are secreted by immune cells as a response to infection and can induce the production of circulating and hypothalamic prostaglandin E2 (PGE2), a final mediator of inflammation-induced thermoregulatory responses [11].

Experimental research on Tb alterations during an infection has widely relied on the use of lipopolysaccharide (LPS), an endotoxin from the cell wall of Gram-negative bacteria, to induce systemic inflammation. This endotoxemia model is advantageous due to its simplicity, dose adjustability, and reproducibility [12]. However, it does not accurately mimic key features of clinical sepsis, that is often caused by Gram-positive bacteria, fungi, or polymicrobial infections [13, 14]. Additionally, LPS triggers a rapid, transient cytokine response, unlike the prolonged inflammation seen in sepsis [15]. Therefore, the results obtained using LPS as a model of systemic inflammation cannot always be directly be extrapolated to sepsis research.

To better understand the pathophysiology of sepsis and improve the potential translational significance of experimental research in the field, we investigated the thermoregulatory responses and underlying mechanisms in a clinically relevant model of experimental sepsis, the cecal ligation and puncture (CLP) method. As a result, we demonstrated that the severity of the disease (mild or severe sepsis) and the ambient temperature (thermoneutral or subthermoneutral) modulate body temperature and the dynamics of thermoeffector recruitment, as well as the outcome of sepsis.

## Materials and methods

### Animals

Male Wistar rats (270 ± 15 g, 8–10 weeks old) from the Animal Care Facility of the University of São Paulo, Campus of Ribeirão Preto, Brazil, were used in the experiments. The animals were individually caged and maintained at a controlled ambient temperature of 23 ± 1 °C and 2 days before the CLP/sham surgery were acclimated at 22 °C (subthermoneutral Ta) or 28 °C (thermoneutral Ta) with a 12-h light/dark cycle with food and water ad libitum. All experiments were approved by the Animal Ethical Committee of the Dental School of Ribeirão Preto (2020.1.391.58.3).

### Cecal ligation and puncture (CLP)

Animals were anesthetized with isoflurane (4% for induction and maintained at 1.5% during the surgery) and the surgical site was shaved with a clipper and disinfected with a 10% povidone-iodine solution. Surgical procedure was performed as described elsewhere [16]. Briefly, a midline abdominal incision was performed using a scalpel blade, the cecum was exposed and it was loosely ligated just above the ileocecal valve. Cecum was perforated four (mild sepsis), seven or ten times (severe sepsis) using a 16G needle. After gentle pressure to allow feces extravasation, the cecum was returned to the peritoneal cavity and the incision was sutured. Saline (10 ml/kg) was injected subcutaneously immediately after surgery. All animals showed typical signs of infection (piloerection, reduced locomotor activity, and tachypnea). Sham animals were submitted to the same procedures, but did not have their cecum ligated or perforated.

### Temperature measurements

To measure core temperature, a datalogger (Subcue, AB, Canada) was inserted in the peritoneal cavity at the moment of the CLP surgery described above. Thermal index (TI) was calculated from the area under the curve during the intervals of time mentioned in each graph. A baseline of 37℃ was set for TI calculation. Skin temperature (Tsk) of the middle third of the tail was measured using an infrared camera (FLIR ONE, USA) every two hours after sepsis induction. The Tsk indicates the peripheral blood flow in rats and is required to infer the heat loss index (HLI). The HLI ranges from 0 to 1 (maximum vasoconstriction and maximum vasodilatation, respectively) and was calculated as HLI = (Tsk -Ta)/(Tb- Ta) [17]. A significant increase or decrease in Tb compared to the respective control animals was considered as fever or hypothermia, respectively.

## Cytokines and PGE2 measurements

Twelve hours after sham/CLP surgery, trunk blood was centrifuged (3500 rpm, 20 min, 4 °C) and plasma was collected for determination of cytokine levels using commercial ELISA kits following the manufacturers’ instructions: IL-1β (DY510, R&D Systems), IL-6 (DY501, R&D Systems) and IL-10 (DY522, R&D Systems). The hypothalamus was dissected and homogenized in RIPA buffer (Sigma-Aldrich, R0278) containing a 10% protease inhibitor cocktail (Sigma-Aldrich, P2714) and 0.5% of phenylmethylsulfonyl fluoride (PMSF). After centrifugation (4300 rpm, 20 min), the supernatant was collected and used to measure PGE2 (514,010; Cayman Chemical) levels following the manufacturers’ instructions. The results were normalized by protein concentration.

### Western blot

Interscapular brown adipose tissue (iBAT) samples we homogenized in RIPA buffer (Sigma-Aldrich, R0278) containing a 10% protease inhibitor cocktail (Sigma-Aldrich, P2714) and 0.5% of phenylmethylsulfonyl fluoride (PMSF), and centrifuged (15,000 RCF, 10 min at 4℃). The supernatant was collected and protein concentration was measured. 30 µg of total protein were separated by electrophoresis in a 12% polyacrylamide gel (50 V for 20 min then 125 V, 120 min) and transferred to a nitrocellulose membrane (100 V, 120 min) in a tank blotting system. Membranes were blocked with 5% skim milk solution in PBS/T 0.5% and incubated overnight at 4℃ under agitation with the primary antibody targeting UCP1 (1:8000, Abcam) and α-tubulin (1:1000, Cell Signaling). After washing 3 × 10 min with PBS/T, membranes were incubated with appropriate HRP-conjugated secondary antibodies (1:10,000 and 1:1000 dilutions, Cell Signaling). Immunoreactive protein bands were visualized using a chemiluminescence reaction kit in the ChemiDoc MP System (BioRad) and analyzed by the system’s software (ImageLab 5.2.1). The image result for each band was normalized against the α-tubulin level (internal control) of the respective sample.

### Statistics

The data are presented as mean ± SD and individual values are presented in the bar graphs. The normality of data was tested using the Shapiro–Wilk normality test. Variables with normal distribution were analyzed by one-way analysis of variance (ANOVA), followed by post hoc Tukey tests. For those with nonparametric distribution, a Kruskal–Wallis test was performed, followed by Dunn’s post hoc tests. The survival rate was expressed as a percentage and a log-rank (Mantel–Cox) test was used to determine differences. Tb results of survivors vs. non-survivors and plasma cytokines data were analyzed using an unpaired t-test. Values of *p* ≤ 0.05 were considered significant.

## Results

### Thermoregulatory responses of septic animals in thermoneutral (28℃) and subthermoneutral (22℃) Ta

**Fig. 1:**
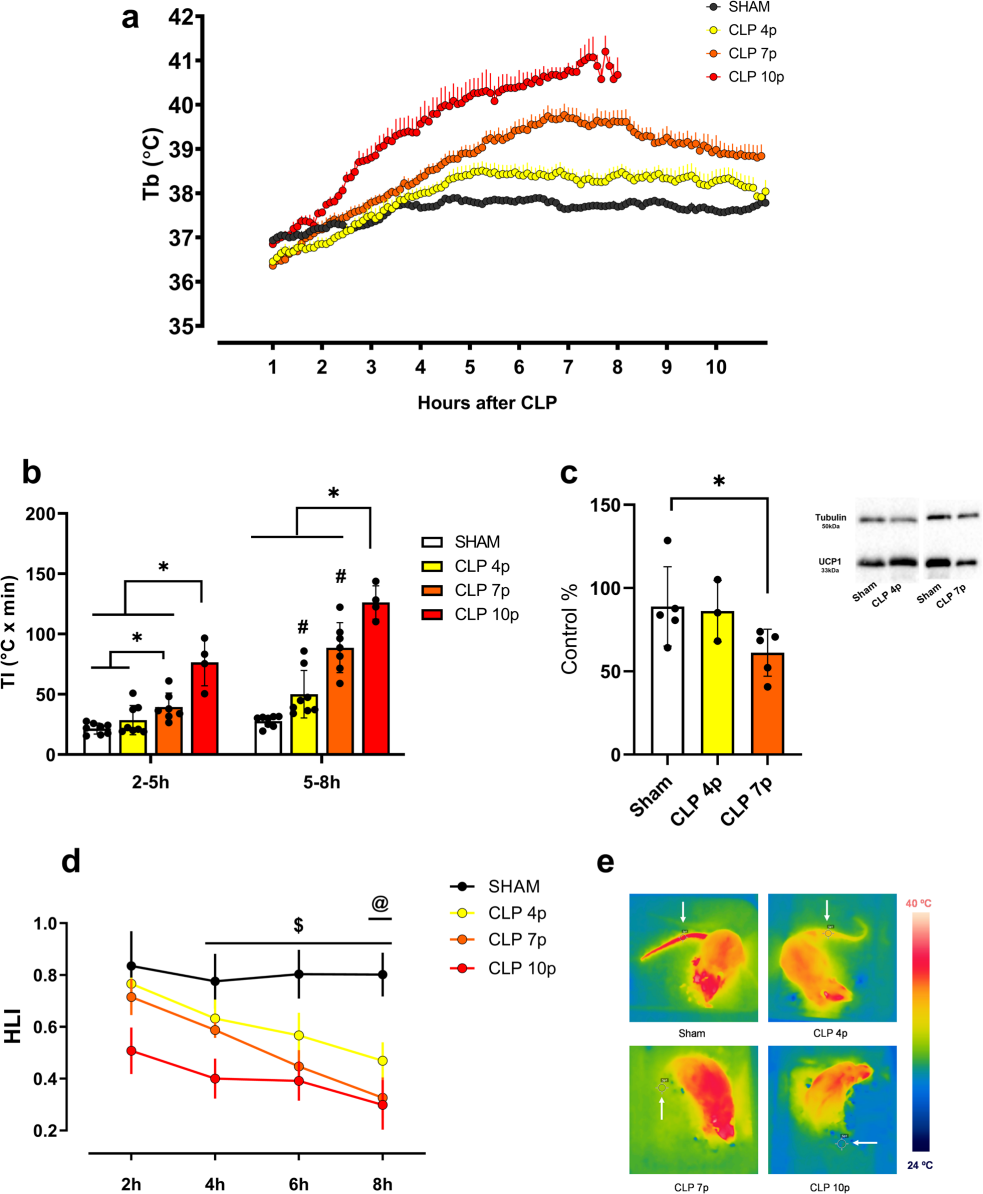
Thermoregulatory responses of septic rats in a thermoneutral ambient temperature (28℃). Body temperature of sham and septic animals subjected to cecal ligation with 4 (CLP 4p—mild sepsis), 7 or 10 punctures (CLP 7p and CLP 10p, respectively—severe sepsis) (sham *n* = 8; CLP 4p *n* = 8; CLP 7p *n* = 7; CLP 10p *n* = 4) (**a**). Thermal index (*area under the curve x time*) of control and septic rats 2–5 h and 5–8 h after surgery (**b**). UCP1 expression in the BAT 12 h after sham or CLP surgery, values are represented as percentage of the control group (**c**). Heat loss index (HLI) variation (sham *n* = 8; CLP 4p *n* = 6; CLP 7p *n* = 7; CLP 10p *n* = 4) (**d**) and representative thermographic images (**e**) obtained 8 h after sepsis, the white arrows point to the Tsk measurement point. *p < 0.05 using one-way ANOVA with Tukey’s or Kruskal–Wallis post hoc test **#** *p* < 0.05 compared to sham group **$** *p* < 0.05 comparing sham vs. CLP 7p and CLP 10p **@** *p* < 0.05 comparing sham vs. CLP 4p groups. Data are presented as mean ± SD. *n* = 4–8

Measurement of body temperature following CLP-induced sepsis in a Ta of 28℃ showed that mild sepsis (4 cecal punctures) caused a moderate and transient increase in Tb, while severe sepsis (7 and 10 punctures) caused a marked and persistent high fever (Fig. 1a). Additionally, severe sepsis (both 7 and 10 punctures) caused an earlier onset of fever compared to mild sepsis (Fig. 1b). At the later stages of sepsis (5-8 h), it was clear that the degree of the fever was corresponding to the severity of the disease (Fig. 1b). Given the high mortality in the CLP 10p group after 10 h (Fig. 3a), we decided to include data up to 8 h after CLP to ensure more accurate comparisons with the other groups.

In order to investigate the thermoeffectors involved in CLP-induced fever, we first accessed the content of UCP1 protein in the iBAT of septic rats as an approach to indirectly evaluate the participation of non-shivering thermogenesis in this process (Fig. 1c). Despite the high fever, our results showed that at 12 h after the CLP surgery, there is a decrease in BAT UCP1 during severe sepsis. However, UCP1 levels were similar in the sham and mild sepsis groups. We then calculated the HLI in order to measure the cutaneous vasomotor activity. The clear decrease of the HLI in the septic groups demonstrates an intense tail vasoconstriction in these animals (Fig. 1d–e).

**Fig. 2:**
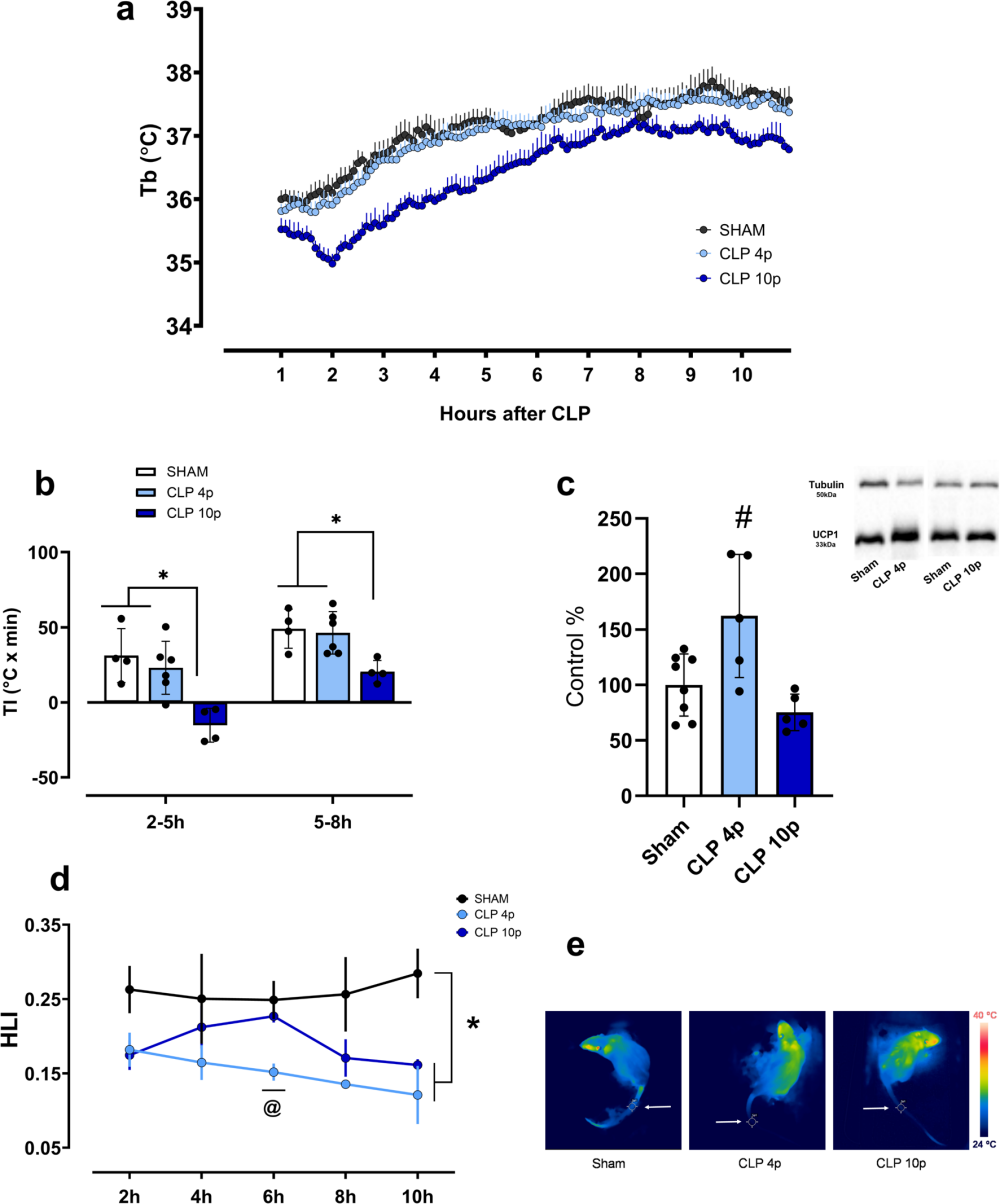
Thermoregulatory responses of septic rats in a subthermoneutral ambient temperature (22℃). Body temperature of sham and septic animals subjected to cecal ligation with 4 (CLP 4p—mild sepsis) or 10 punctures (CLP 10p—severe sepsis) (sham *n* = 4; CLP 4p *n* = 6; CLP 10p *n* = 4) (**a**). Thermal index (*area under the curve x time*) of control and septic rats 2–5 h and 5–8 h after surgery (**b**). UCP1 expression in the BAT 12 h after sham or CLP surgery, values are represented as percentage of the control group (**c**). Heat loss index (HLI) variation (sham *n* = 4; CLP 4p *n* = 6; CLP 10p *n = *4) (**d**) and representative thermographic images (**e**) obtained 10 h after sepsis, the white arrows point to the Tsk measurement point. *p < 0.05 using one-way ANOVA with Tukey’s or Kruskal–Wallis post hoc test **#** *p* < 0.05 compared to sham group **@** *p* < 0.05 comparing sham vs. CLP 4p groups. Data are presented as mean ± SD. *n* = 4–8

Rats subject to sepsis in an ambient temperature of 22℃ displayed distinct changes in body temperature compared to those at 28℃. When housed in a subthermoneutral ambient, animals subjected to mild sepsis did not show any alteration in Tb compared to sham animals, while a long-lasting lower Tb was observed within one hour from the onset of severe sepsis (10 punctures) (Fig. 2a, b). In addition, UCP1 expression in BAT was significantly increased 12 h after mild sepsis, but not in the 10-punctures CLP group (Fig. 2c). As indicated by HLI values, during mild sepsis there is an early reduction in tail heat loss (at 6 h), while rats subjected to severe sepsis only showed a statistically significant difference when compared to sham animals at 10 h post-sepsis (Fig. 2d, e).

### Effect of sepsis severity and ambient temperature on mortality

**Fig. 3:**
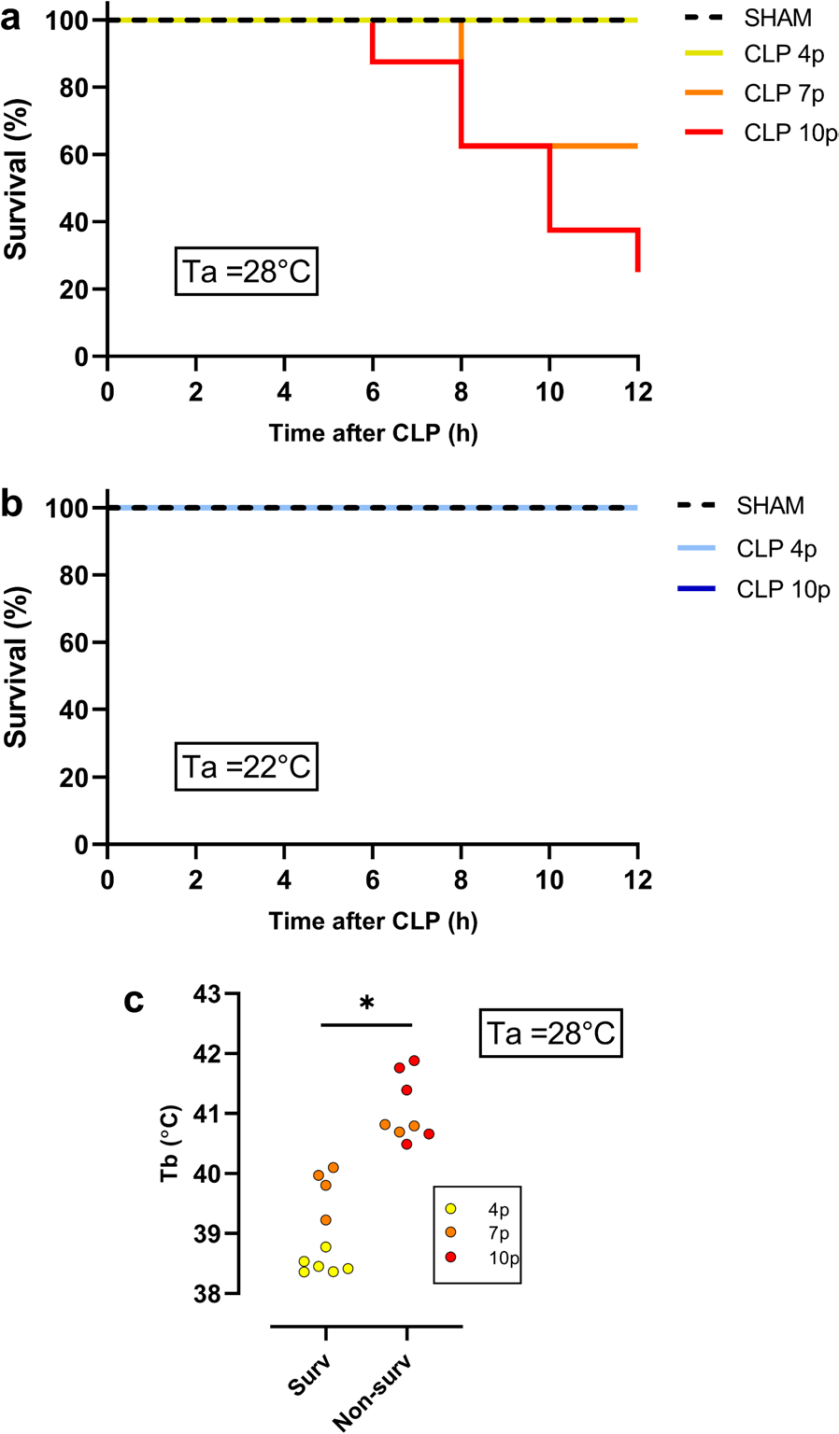
Survival rate 12 hours after sepsis in different ambient temperatures. Survival curve of animals subjected to mild (CLP 4p) and severe (CLP 7p and CLP 10p) sepsis in a thermoneutral (sham *n* = 8; CLP 4p *n* = 8; CLP 7p *n* = 8; CLP 10p *n* = 7) (**a**) and subthermoneutral (sham *n* = 4; CLP 4p *n* = 6; CLP 10p *n* = 5) (**b**) ambient temperature. **c** Values of the maximum body temperature of survivors (*surv*) versus non-survivor (*non-surv*) septic rats housed at 28℃ *p < 0.05 using a non-paired t-test. Data are presented as mean ± SD. *n* = 8–10

There is a long-lasting debate regarding body temperature in septic patients, trying to elucidate whether Tb is related to certain clinical outcome and mortality rate [18, 19]. At a Ta of 28 ℃, in mild sepsis (4 punctures during the CLP) there was no death in a 12-h period, while rats subjected to 7 punctures or 10 punctures during CLP had a survival of 40% and 20%, respectively, during the same time frame (Fig. 3a). We also observed a clear difference in the maximum Tb reached by non-survivors compared to survivors (Fig. 3c), showing that non-survivors had a higher fever. Interestingly, there was not an overlap of the maximum Tb between the groups: the highest Tb of the survivors had a range of 38.3–40.1 ℃, while the non-survivors had a range of Tb between 40.5 and 41.9 ℃. Given the high mortality of the group 10 punctures at 28℃, we performed posterior analysis (PGE2 and cytokines measurements) only in the 7-punctures group and considered it as a model of severe sepsis. At a temperature of 22 ℃ it was not observed any death in septic animals, independently of the severity of the disease (Fig. 3b).

### Hypothalamic PGE2 and systemic cytokine synthesis during CLP-induced sepsis

**Fig. 4:**
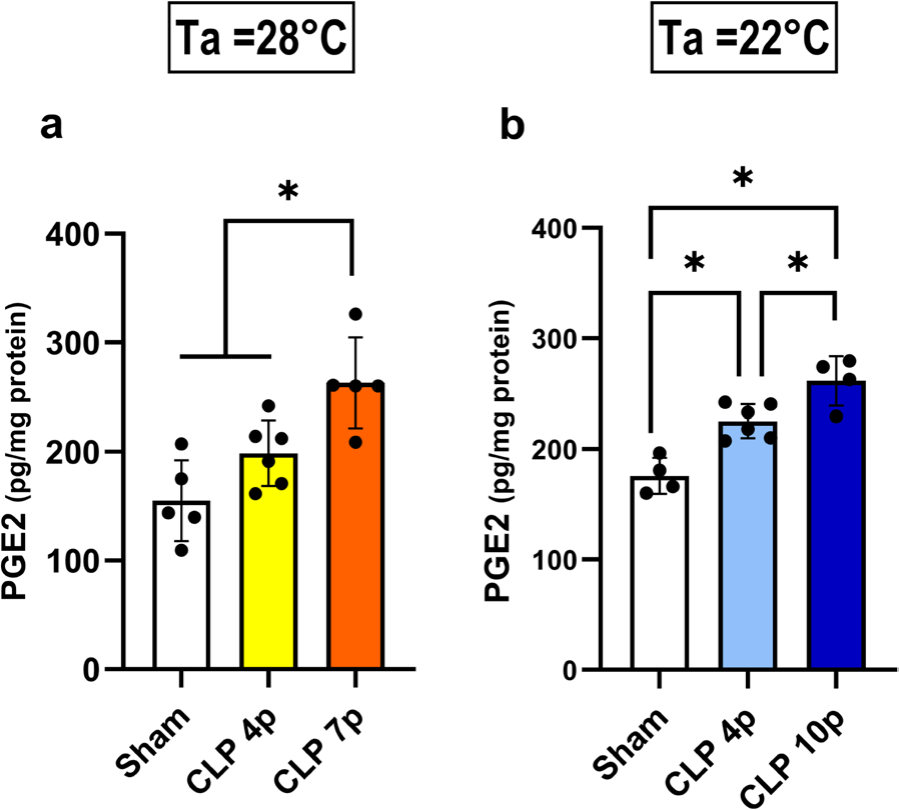
PGE2 synthesis in the hypothalamus. Hypothalamic concentration (pg/mg protein) of PGE2 of septic and control animals in a thermoneutral (**a**) or subthermoneutral (**b**) ambient temperature. Samples were collected 12 hours after CLP/ sham surgeries. Data are presented as mean ± SD. *p < 0.05 using one-way ANOVA. *n* = 4–6

PGE2 synthesis in the preoptic area of the hypothalamus has a prominent role in the regulation of body temperature during an infection [20]. In our model, sepsis led to an increase in hypothalamic PGE2 levels, regardless of the ambient temperature, except in mild sepsis at Ta = 28℃ (Fig. 4). Furthermore, PGE2 levels were higher in severe sepsis (10p) compared to mild sepsis (4p) at both Ta.

**Fig. 5:**
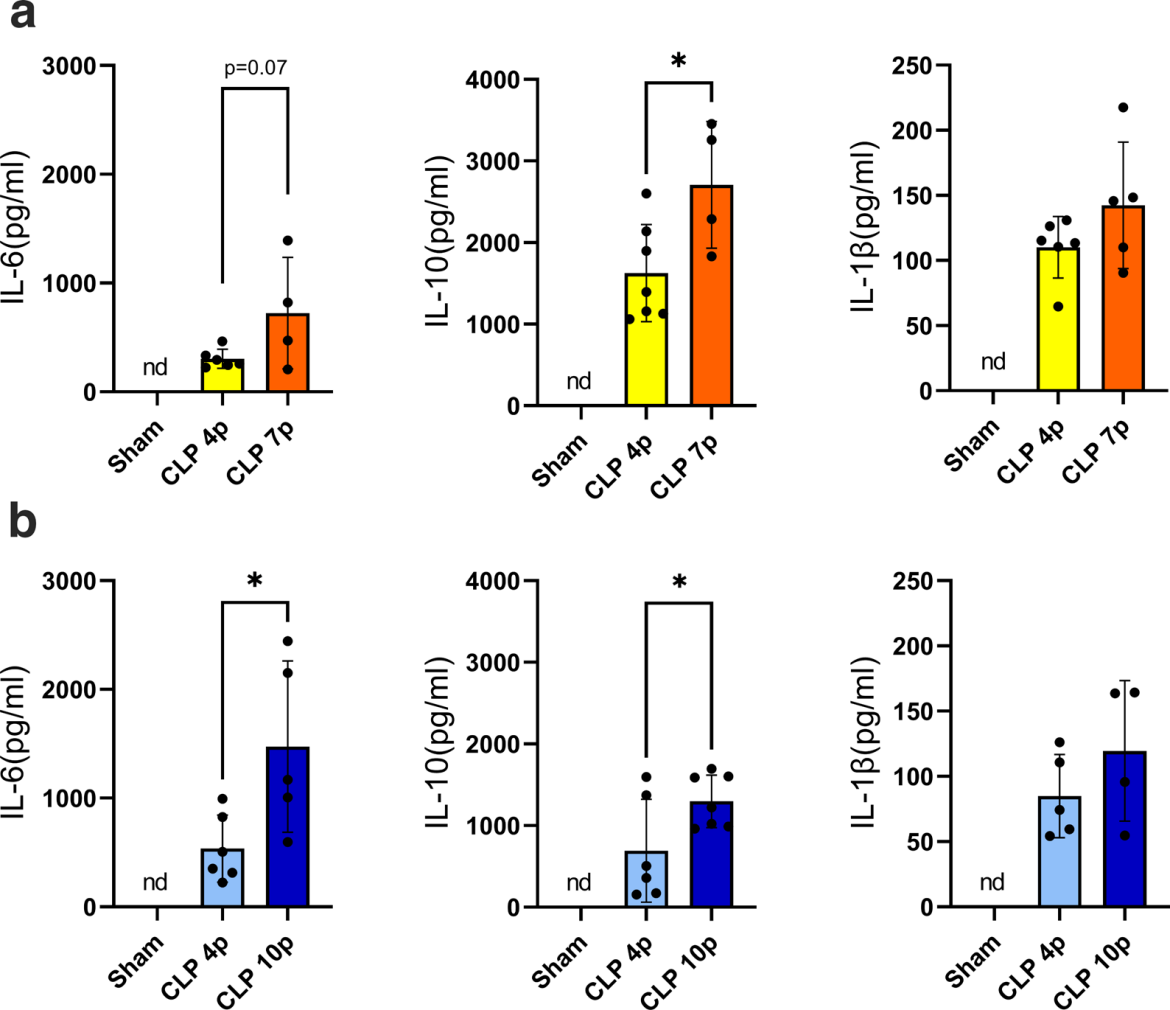
Plasma inflammatory mediators during sepsis. Circulating levels of IL-6, IL-10 and IL-1β in septic and control rats in a thermoneutral (**a**) or subthermoneutral (**b**) ambient temperature. nd = nondetectable. Data are presented as mean ± SD. *p < 0.05 using a non-paired t-test. *n* = 4–7

Systemic inflammation during sepsis is characterized by the marked production of several inflammatory mediators, the so called “cytokine storm”. In our experiments we found an increase in the circulating levels of inflammatory cytokines (IL-6, IL-10, IL-1β) in response to CLP, both at 22℃ and 28℃ (Fig. 5 a-b). We also observed a more prominent inflammatory response in severe sepsis than in mild sepsis, with higher levels of IL-6 and IL-10 in 7 or 10-punctures CLP than in 4-punctures CLP (Fig. 5).

## Discussion

In this study, we investigated how both ambient temperature and infection severity affect body temperature in animals subjected to cecal ligation and puncture-induced sepsis, a clinically relevant experimental model of this disease. Our findings suggest that at thermoneutrality (28℃ for rats), sepsis is associated with the development of fever, increase of plasma cytokine levels, and elevated hypothalamic PGE_2_ production and the mortality rate of animals was proportional to the severity of the disease. Conversely, in a cooler environment (Ta = 22 ℃), septic rats either maintained their Tb during mild sepsis or developed hypothermia during severe sepsis, with no observed mortality, despite the marked inflammatory response. We also demonstrate that the hability to regulate Tb, through mechanisms like tail skin vasoconstriction and brown adipose tissue thermogenesis, is influenced by both ambient temperature and the severity of sepsis. Specifically, HLI was reduced in all septic animals, regardless of Ta, while UCP-1 expression in brown adipose tissue increased only in mild sepsis under subthermoneutral conditions.

Despite the extensive research in experimental sepsis, there are several unsolved questions about the thermoregulatory dynamics during this disease. Body temperature was accessed in previous studies using the CLP method and both fever [21–23] and hypothermia [24–26] were observed, but none of these publications focused on the intrinsic body mechanisms associated with the changes in Tb and their relation with Ta.

Using the method of Romanovsky et al*.*, which is based on the fact that rats have moderate tail skin vasodilatation in a neutral environment, we considered that Ta = 28 °C was thermoneutral and Ta = 22 °C was subthermoneutral in our experimental protocol. The thermoneutral zone (TMZ) consists of the range of Ta at which temperature regulation is achieved only by controlling heat loss and not by changes in metabolism [17]. At 28 °C, independently of sepsis severity, a fever response was observed, associated with a reduction of heat loss by the tail. It is also important to consider that the fever response might be influenced by the thermal gradient at this temperature, potentially making heat retention more effective. As BAT UCP1 expression was not altered in mild sepsis and had a decrease after severe sepsis, we infer that fever was mediated by tail vasoconstriction rather than an increase in non-shivering thermogenesis. Although we did not measure oxygen consumption as an index of non-shivering thermogenesis, a previous report corroborates our results, showing that CLP does not increase the expression of genes related to thermogenesis in the BAT, including *Ucp1*gene [26]. We speculate that cytokines such as IL-6, which has a thermogenic effect in chronic situations [27, 28], reduces BAT thermogenesis in acute conditions like sepsis due to its potent proinflammatory effects [29, 30]. These observations support the hypothesis that BAT thermogenesis is not the underlying mechanism for fever in our CLP model.

Similarly to what is observed in studies using different doses of LPS in a thermoneutral ambient [31], animals responded to sepsis with fever in a severity-dependent fashion. The degree of the increase in Tb can be also associated to the level of the inflammatory response and PGE2 synthesis. While circulating PGE2 produced by immune cells seems to be an initiator of fever, brain PGE2 is responsible for sustaining it [32]. In the hypothalamus, PGE2 produced by microglia and endothelial cells binds to EP3 receptors, an inhibitory receptor, expressed in glutamatergic neurons in the preoptic area which ultimately induces fever response [33, 34]. Supporting the febrigenic role of PGE2 in sepsis, it has been already shown that the administration of celecoxib, a selective COX-2 inhibitor, inhibited febrile response in septic rats after CLP [22]. Despite the importance of PGE2 in producing fever responses, fever observed during mild sepsis may be also result of a prostaglandin-independent pathway mediated by IL-1β [35]. A limitation of our study is the time point we analyzed the PGE2 levels, as PGE2 may be elevated earlier during mild sepsis but not after 12 h.

Fever is considered a defensive mechanism during an infection because it stimulates immune function and directly inhibits pathogens [36], but an exacerbated response can be deleterious to the organism. Dramatic increases in body temperature can lead to cell death mainly through the disruption of membrane stability and transmembrane transport, and the impairment of protein and DNA synthesis [37, 38]. In our experiments, an increased fever response was associated with higher mortality. Interestingly there was not an overlap of the peak of Tb (independently of the severity of sepsis) among the survivors (40.1 °C maximum) compared to non-survivors (40.5℃ or higher), suggesting that a point/range of Tb between these values is the highest temperature tolerated by septic rats. However, it is challenging to determine whether fever was the direct cause of death or a symptom of the harmful inflammatory response. It is likely that a combination of both factors contributed to organ dysfunction and subsequent death. The liver, for instance, is significantly impacted during sepsis, and markers of hepatic dysfunction are associated with mortality in the CLP model [39]. Previous studies have indicated that cytokines impair liver function during systemic inflammation [40, 41], but it has also been demonstrated that hyperthermia itself can be detrimental by reducing blood flow to the liver and causing hypoxia-induced hepatic damage [42]. Markers of heart [43, 44], kidney [45] and brain [46, 47] dysfunction are all increased during sepsis, even in the early stages of the disease, and are correlated to increased mortality.

When housed in a subthermoneutral environment (22℃), septic rats subjected to mild sepsis did not show changes in Tb, while those under severe sepsis had significant hypothermia. These findings are comparable to a study showing that the administration of the same dose of LPS provoked opposite changes in Tb when animals were in different ambient temperatures (fever in a thermoneutral Ta and hypothermia in a cool Ta) [31]. Thermoeffector recruitment was also distinct from a thermoneutral ambient. During mild sepsis, we observed a more consistent tail vasoconstriction and an increase in UCP1 expression in the BAT. The concomitant inhibition of heat loss and the increase of heat generation might have contributed to maintaining Tb at basal levels throughout the whole period of time analyzed, despite the substantially increased inflammatory response. On the other hand, in severe sepsis, UCP1 levels did not change and HLI was reduced only 10 h after CLP, which potentially contributed to the reduction in body temperature. These results point to a complex intercommunication between ambient temperature, infection severity, and thermoeffector recruitment. Interestingly, and aligned with our results, a study identified that lower ambient temperature was an independent predictor of hypothermia in septic patients [48].

Our data can be examined in light of an organism's dual protective strategies, i.e., resistance and tolerance, when facing infection, and how these strategies are influenced by Ta [49, 50]. Resistance involves an acute cellular immune response that actively attacks the pathogen, incurring significant metabolic costs. Tolerance, in contrast, suppresses the self-reactive immune response to limit tissue damage, thereby conserving energy for other physiological functions. A recent study [51] highlights this complexity showing that bacterial infection or LPS causes a trade-off with homeothermy and that infectious stress (at 22℃) induces hypothermia as an energy-conserving state. Our findings indicate that during severe sepsis, the host's response is distinctly modulated by Ta. At thermoneutral Ta, the organism favors a resistance strategy, leading to a dynamic but metabolically expensive immune response that increases mortality. Conversely, in a subthermoneutral Ta, the additional metabolic demand for BAT thermogenesis promotes a shift towards tolerance. This hypometabolic-hypothermic state is protective, reducing the deleterious effects of an overactive immune response. Interestingly, in mild sepsis, animals successfully controlled the infection regardless of Ta, evidenced by 100% survival. These results highlight the critical role of energetic trade-offs in determining the host's immune strategy, emphasizing the intricate crosstalk between homeothermy and immune function in managing energy consumption and optimizing survival outcomes during infection.

Previous studies have shown that metabolic stressors can significantly impact sepsis outcomes. For instance, Starr et al. [52] demonstrated that dietary restriction can improve survival rates in septic models by modulating Tb and the immune response, reducing inflammation. Other metabolic disorders like obesity [53, 54] and diabetes [55] also impact sepsis outcomes. The investigation of the impact of these circumstances during sepsis could provide valuable insights into the mechanisms of disease tolerance and resistance. Further research is needed to elucidate the underlying mechanisms of this crosstalk and its potential therapeutic implications for managing infectious diseases.

Despite the clinical significance of hypothermia during systemic inflammation, our understanding of its pathophysiology and the underlying mechanisms remains very limited compared to fever. PGE2, which has been extensively associated with fever, was also increased in the hypothalamus of hypothermic animals, probably as a consequence of the increased inflammatory response caused by CLP. Four subtypes of PGE2 (EP) receptors have been identified and while the activation of EP1 or EP3 receptors increases body temperature, administration of a EP4 receptor agonist decreases Tb [56]. The dynamics of the interplay between different prostaglandin receptors during sepsis and how it can lead to fever or hypothermia remains to be deciphered. We speculate that other mediators could also play a role in sepsis-induced hypothermia. Previous studies have shown evidence that prostaglandin D2 (PGD2), for example, is involved in hypothermic responses after LPS injection [57, 58].

We reinforce that we do not aim to suggest a duality between *good vs. bad* body or ambient temperatures during sepsis. Despite recent advances in the investigation of Tb changes and the outcome of sepsis, there is still no consensus if fever or hypothermia would result in a better prognostic in patients [59, 60]. Previous studies have reported different results from ours, with lower mortality rates after CLP in animals housed at thermoneutral Ta [61] and indicating that lower Ta exacerbates neuroinflammation [62] and cardiac dysfunction [63] in models of endotoxemia. Our data, however, point out that hypothermia can be protective during severe sepsis. Although it is often perceived as a thermoregulatory failure of the organism during an infection, it has been shown that Tb reduction in septic patients is a transient and non-terminal response [59]. Additionally, experimental studies have shown that this is a regulated process, i.e., a physiologically controlled phenomenon just like fever [64, 65]. These contradictory results highlight the complexity of this field of investigation and may be explained by differences in experimental methods. Variations can include the model of systemic inflammation used (such as CLP or LPS), the severity of the immune challenge (e.g., dose of LPS, number/ size of cecal punctures), the timepoint of analysis (early versus late phases of infection), the animal species (rats or mice), among other factors (sex, age, etc.). Translationally, these conflicting results seem to suggest that stratification of septic patients (by age, disease severity or metabolic status, for example) could be a better approach to investigate if cooling or warming would be a beneficial therapeutic effect.

Our investigation has some limitations. We only analyzed thermoregulatory alterations during the early stage of sepsis, specifically within 12 h after CLP. Although a previous study indicated that septic animals return their body temperature to basal levels 24 h even after severe CLP [22], it would be valuable to investigate how thermoeffectors are recruited and how Ta impacts survival in the later phases of infection. Additionally, we did not evaluate the disease progression across different severities and ambient temperatures over time. Implementing a scoring system to access the evolution of sepsis in our experiments could provide more comprehensive data to associate with mortality outcomes.

Finally, we would like to highlight the findings by Helbing et al. [66] where the authors reviewed recent publications (2019–2022) regarding sepsis research and observed a notable disregard of ambient temperature, which is often not even specified in publications. Our results revealed that changes in Ta may lead to completely different physiological responses and alter mortality during sepsis, and thus is relevant variable to be considered in experimental studies. In order to have comparable results and consistent findings we highly recommend that investigators always control and specify the ambient temperature in their protocols and publications when using experimental models of systemic inflammation.

### Perspectives and conclusion

Our results indicate that the thermoregulatory mechanisms activated during sepsis are highly dependent on the severity of the condition and the surrounding environment. For instance, the protective role of hypothermia observed in severe sepsis within a subthermoneutral environment suggests a potential adaptive mechanism that could be therapeutically harnessed. This finding supports the growing interest in using hypothermia as a therapeutic strategy in the clinical practice [67–69]. Moreover, our data suggest that thermoregulatory responses, particularly the dynamics of fever and hypothermia, could serve as important biomarkers for assessing sepsis severity and prognosis. This could pave the way for developing more refined diagnostic tools that incorporate body temperature patterns as part of the criteria for stratifying sepsis patients, potentially leading to more personalized treatment approaches. Additionally, we emphasize the importance of considering ambient temperature in modulating immune response and survival outcomes during sepsis, underscoring the necessity of accounting for environmental factors in clinical settings.

Our results also demonstrate the necessity of accurately controlling ambient temperature in preclinical investigations. The current version of the *Guide for the Care and Use of Laboratory Animals* by the National Research Council in the USA, for example, recommends a housing temperature of 20–26 °C for mice and rats, a range that is below their thermoneutral zone. Prolonged exposure to a cold environment not only increases energy expenditure, but also leads to an increase in norepinephrine (NE) release, which acts on BAT to induce thermogenesis. However, NE's actions on the organism are not confined to BAT and have systemic effects, including on immune cells [70]. Chronic stress is associated with worse outcomes in autoimmune diseases and low-grade inflammation, for example [71, 72]. These changes in immune function should be carefully considered during experimental sepsis research. The impact of the length of exposure to a subthermoneutral temperature prior to infection has not been explored, but understanding this could provide important information regarding the necessary acclimation period for animals.

In conclusion, our experimental results have significant implications for the conceptual understanding of sepsis pathophysiology and translational relevance for diagnosis and therapy. By embedding our findings within the broader context of sepsis research, we demonstrate that both environmental and metabolic factors play critical roles in the pathophysiology of sepsis. This integrated approach not only advances our knowledge of the disease mechanisms, but also paves the way for developing innovative diagnostic and therapeutic strategies that could benefit a broader audience, including both clinical practitioners and researchers.

## Data Availability

The datasets used and/or analyzed during the current study are available from the corresponding author on reasonable request.

## Abbreviations

ATP: Adenosine triphosphate
BAT: Brown adipose tissue
CLP: Cecal ligation and puncture
COX-2: Cyclooxygenase-2
HLI: Heat loss index
ICU: Intensive care unit
IL: Interleukin
LPS: Lipopolysaccharide
NE: Norepinephrine
PGE2: Prostaglandin E2
Ta: Ambient temperature
Tb: Body temperature
TMZ: Thermoneutral zone
TNF: Tumor necrosis factor
UCP1: Uncoupling protein 1

## References

[1] Tan CL, Knight ZA (2018) Regulation of body temperature by the nervous system. Neuron 98:31 29621489 10.1016/j.neuron.2018.02.022 PMC6034117

[2] Branco LGS, Soriano RN, Steiner AA (2014) Gaseous mediators in temperature regulation. Compr Physiol. 10.1002/cphy.c130053 25428845 10.1002/cphy.c130053

[3] Ishiwata T, Hasegawa H, Yasumatsu M et al (2001) The role of preoptic area and anterior hypothalamus and median raphe nucleus on thermoregulatory system in freely moving rats. Neurosci Lett. 10.1016/S0304-3940(01)01865-1 11403973 10.1016/s0304-3940(01)01865-1

[4] Boulant JA (2000) Role of the preoptic-anterior hypothalamus in thermoregulation and fever. Clin Infect Dis. 10.1086/317521 11113018 10.1086/317521

[5] Rand RP, Burton AC, Ing T (1965) The tail of the rat, in temperature regulation and acclimatization. Can J Physiol Pharmacol. 10.1139/y65-025 14329334 10.1139/y65-025

[6] Gordon CJ (1990) Thermal biology of the laboratory rat. Physiol Behav 47:963 2201986 10.1016/0031-9384(90)90025-y

[7] Fedorenko A, Lishko PV, Kirichok Y (2012) Mechanism of fatty-acid-dependent UCP1 uncoupling in brown fat mitochondria. Cell. 10.1016/j.cell.2012.09.010 23063128 10.1016/j.cell.2012.09.010 PMC3782081

[8] Cannon B, Nedergaard J (2004) Brown adipose tissue: function and physiological significance. Physiol Rev 84:277 14715917 10.1152/physrev.00015.2003

[9] Atkins E (1982) Fever: Its history, cause, and function. Yale J Biol Med 55:283 6758374 PMC2596465

[10] Bone RC, Balk RA, Cerra FB et al (1992) Definitions for sepsis and organ failure and guidelines for the use of innovative therapies in sepsis. Chest. 10.1378/chest.101.6.1644 1303622 10.1378/chest.101.6.1644

[11] Steiner AA, Branco LGS (2003) Fever and anapyrexia in systemic inflammation: intracellular signaling by cyclic nucleotides. Front Biosci 8:s1398 12957844 10.2741/1188

[12] Lewis AJ, Seymour CW, Rosengart MR (2016) Current murine models of sepsis. Surg Infect. 10.1089/sur.2016.021 10.1089/sur.2016.021 PMC4960474 27305321

[13] Martin GS, Mannino DM, Eaton S, Moss M (2003) The epidemiology of sepsis in the United States from 1979 through 2000. New Eng J Med. 10.1056/nejmoa022139 12700374 10.1056/NEJMoa022139

[14] Magill SS, Sapiano MRP, Gokhale R et al (2023) Epidemiology of sepsis in US children and young adults. Open Forum Infect Dis. 10.1093/ofid/ofad218 37187509 10.1093/ofid/ofad218 PMC10167985

[15] Zhang B, Liu C, Yang N, Wang X (2017) A comparative study of changes of autophagy in rat models of CLP versus LPS induced sepsis. Exp Ther Med. 10.3892/etm.2017.4758 28962141 10.3892/etm.2017.4758 PMC5609131

[16] da Costa LHA, Santos-Junior NN, Catalão CHR, Rocha MJA (2021) Microglial activation modulates neuroendocrine secretion during experimental sepsis. Mol Neurobiol. 10.1007/s12035-020-02241-5 33415683 10.1007/s12035-020-02241-5

[17] Romanovsky AA, Ivanov AI, Shimansky YP (2002) Selected contribution: Ambient temperature for experiments in rats: a new method for determining the zone of thermal neutrality. J Appl Physiol. 10.1152/japplphysiol.01173.2001 12015388 10.1152/japplphysiol.01173.2001

[18] Rumbus Z, Garami A (2019) Fever, hypothermia, and mortality in sepsis. Temperature. 10.1080/23328940.2018.1516100 10.1080/23328940.2018.1516100 PMC6601416 31286020

[19] Steiner AA, Fonseca MT, Soriano FG (2017) Should we assume that hypothermia is a dysfunction in sepsis? Crit Care. 10.1186/s13054-016-1584-y 28073371 10.1186/s13054-016-1584-y PMC5225576

[20] Roth J, Rummel C, Barth SW et al (2009) Molecular aspects of fever and hyperthermia. Immunol Allergy Clin North Am 29:229 19389579 10.1016/j.iac.2009.02.005

[21] Leon LR, White AA, Kluger MJ (1998) Role of IL-6 and TNF in thermoregulation and survival during sepsis in mice. Am J Physiol Regul Integr Comp Physiol. 10.1152/ajpregu.1998.275.1.r269 10.1152/ajpregu.1998.275.1.R269 9688988

[22] Figueiredo MJ, De Melo SD, Martins JM et al (2012) Febrile response induced by cecal ligation and puncture (CLP) in rats: Involvement of prostaglandin E2 and cytokines. Med Microbiol Immunol. 10.1007/s00430-011-0225-y 22203392 10.1007/s00430-011-0225-y

[23] Passaglia P, Silva HB, de Jesus AA et al (2023) Angiotensin-(1–7) improves tail skin heat loss and increases the survival of rats with polymicrobial sepsis. Peptides. 10.1016/j.peptides.2023.171042 37315714 10.1016/j.peptides.2023.171042

[24] Granger JI, Ratti PL, Datta SC et al (2013) Sepsis-induced morbidity in mice: Effects on body temperature, body weight, cage activity, social behavior and cytokines in brain. Psychoneuroendocrinology. 10.1016/j.psyneuen.2012.10.010 23146654 10.1016/j.psyneuen.2012.10.010 PMC3707484

[25] Mai SHC, Sharma N, Kwong AC et al (2018) Body temperature and mouse scoring systems as surrogate markers of death in cecal ligation and puncture sepsis. Intensive Care Med Exp. 10.1186/s40635-018-0184-3 30054760 10.1186/s40635-018-0184-3 PMC6063809

[26] Moreno-Navarrete JM, Comas F, de Jager V et al (2021) Cecal ligation and puncture-induced sepsis promotes brown adipose tissue inflammation without any impact on expression of thermogenic-related genes. Front Physiol. 10.3389/fphys.2021.692618 34322037 10.3389/fphys.2021.692618 PMC8313297

[27] Dong M, Gao C, Jia Y et al (2022) Temporal specificity of IL-6 knockout in enhancing the thermogenic capability of brown adipose tissue. J Physiol Biochem. 10.1007/s13105-021-00847-4 35349101 10.1007/s13105-021-00847-4

[28] Egecioglu E, Anesten F, Schéle E, Palsdottir V (2018) Interleukin-6 is important for regulation of core body temperature during long-term cold exposure in mice. Biomed Rep. 10.3892/br.2018.1118 30271595 10.3892/br.2018.1118 PMC6158403

[29] Omran F, Christian M (2020) Inflammatory signaling and brown fat activity. Front Endocrinol. 10.3389/fendo.2020.00156 10.3389/fendo.2020.00156 PMC7105810 32265845

[30] Sakamoto T, Nitta T, Maruno K et al (2016) Macrophage infiltration into obese adipose tissues suppresses the induction of UCP1 level in mice. Am J Physiol Endocrinol Metab. 10.1152/ajpendo.00028.2015 26884382 10.1152/ajpendo.00028.2015

[31] Rudaya AY, Steiner AA, Robbins JR et al (2005) Thermoregulatory responses to lipopolysaccharide in the mouse: dependence on the dose and ambient temperature. Am J Physiol Regul Integr Comp Physiol. 10.1152/ajpregu.00370.2005 16081879 10.1152/ajpregu.00370.2005

[32] Steiner AA, Ivanov AI, Serrats J et al (2006) Cellular and molecular bases of the initiation of fever. PLoS Biol. 10.1371/journal.pbio.0040284 16933973 10.1371/journal.pbio.0040284 PMC1551923

[33] Machado NLS, Bandaru SS, Abbott SBG, Saper CB (2020) Ep3R-expressing glutamatergic preoptic neurons mediate inflammatory fever. J Neurosci. 10.1523/JNEUROSCI.2887-19.2020 32079648 10.1523/JNEUROSCI.2887-19.2020 PMC7083539

[34] Lazarus M, Yoshida K, Coppari R et al (2007) EP3 prostaglandin receptors in the median preoptic nucleus are critical for fever responses. Nat Neurosci. 10.1038/nn1949 17676060 10.1038/nn1949

[35] Mota CMD, Madden CJ (2022) Systemic Interleukin-1β elicits cyclooxygenase-independent fever. FASEB J. 10.1096/fasebj.2022.36.s1.0r796

[36] Wrotek S, Legrand EK, Dzialuk A, Alcock J (2021) Let fever do its job. Evol Med Public Health 9:26 33738101 10.1093/emph/eoaa044 PMC7717216

[37] Walter EJ, Hanna-Jumma S, Carraretto M, Forni L (2016) The pathophysiological basis and consequences of fever. Crit Care. 10.1186/s13054-016-1375-5 27411542 10.1186/s13054-016-1375-5 PMC4944485

[38] Hildebrandt B, Wust P, Ahlers O et al (2002) The cellular and molecular basis of hyperthermia. Crit Rev Oncol Hematol 43:33 12098606 10.1016/s1040-8428(01)00179-2

[39] Drechsler S, Weixelbaumer KM, Weidinger A et al (2015) Why do they die? Comparison of selected aspects of organ injury and dysfunction in mice surviving and dying in acute abdominal sepsis. Intensive Care Med Exp. 10.1186/s40635-015-0048-z 26215812 10.1186/s40635-015-0048-z PMC4513036

[40] Bode JG, Albrecht U, Häussinger D et al (2012) Hepatic acute phase proteins—regulation by IL-6- and IL-1-type cytokines involving STAT3 and its crosstalk with NF-κB-dependent signaling. Eur J Cell Biol 91:496 22093287 10.1016/j.ejcb.2011.09.008

[41] Strnad P, Tacke F, Koch A, Trautwein C (2017) Liver-guardian, modifier and target of sepsis. Nat Rev Gastroenterol Hepatol 14:55 27924081 10.1038/nrgastro.2016.168

[42] Thorne AM, Ubbink R, Bruggenwirth IMA et al (2020) Hyperthermia-induced changes in liver physiology and metabolism: a rationale for hyperthermic machine perfusion. Am J Physiol Gastrointest Liver Physiol 319:G43 32508156 10.1152/ajpgi.00101.2020

[43] Shvilkina T, Shapiro N (2023) Sepsis-Induced myocardial dysfunction: heterogeneity of functional effects and clinical significance. Front Cardiovasc Med. 10.3389/fcvm.2023.1200441 37522079 10.3389/fcvm.2023.1200441 PMC10375025

[44] Zhang L, Qi D, Peng M et al (2023) Decoding molecular signature on heart of septic mice with distinct left ventricular ejection fraction. iScience. 10.1016/j.isci.2023.107825 37736036 10.1016/j.isci.2023.107825 PMC10509301

[45] Stanski NL, Cvijanovich NZ, Fitzgerald JC et al (2020) Severe acute kidney injury is independently associated with mortality in children with septic shock. Intensive Care Med 46:1050 32047942 10.1007/s00134-020-05940-8 PMC7677896

[46] Schuler A, Wulf DA, Lu Y et al (2018) The impact of acute organ dysfunction on long-term survival among sepsis survivors HHS public access. Crit Care Med 46:1050 10.1097/CCM.0000000000003023 PMC5953770 29432349

[47] Ritter C, Miranda AS, Giombelli VR et al (2012) Brain-derived neurotrophic factor plasma levels are associated with mortality in critically ill patients even in the absence of brain injury. Crit Care. 10.1186/cc11902 23245494 10.1186/cc11902 PMC3672623

[48] Thomas-Rüddel DO, Hoffmann P, Schwarzkopf D et al (2021) Fever and hypothermia represent two populations of sepsis patients and are associated with outside temperature. Crit Care. 10.1186/s13054-021-03776-2 34674733 10.1186/s13054-021-03776-2 PMC8532310

[49] McCarville JL, Ayres JS (2018) Disease tolerance: concept and mechanisms. Curr Opin Immunol 50:88 29253642 10.1016/j.coi.2017.12.003 PMC5884632

[50] Soares MP, Teixeira L, Moita LF (2017) Disease tolerance and immunity in host protection against infection. Nat Rev Immunol 17:83 28044057 10.1038/nri.2016.136

[51] Ganeshan K, Nikkanen J, Man K et al (2019) Energetic trade-offs and hypometabolic states promote disease tolerance. Cell. 10.1016/j.cell.2019.01.050 30853215 10.1016/j.cell.2019.01.050 PMC6456449

[52] Starr ME, Steele AM, Cohen DA, Saito H (2016) Short-term dietary restriction rescues mice from lethal abdominal sepsis and endotoxemia and reduces the inflammatory/coagulant potential of adipose tissue. Crit Care Med. 10.1097/CCM.0000000000001475 26646465 10.1097/CCM.0000000000001475 PMC4896861

[53] Gao L, Jin LJ, Chao FQ et al (2023) Association of obesity and mortality in sepsis patients: a meta-analysis from observational evidence. Heliyon. 10.1016/j.heliyon.2023.e19556 37809532 10.1016/j.heliyon.2023.e19556 PMC10558781

[54] Bai L, Huang J, Wang D et al (2023) Association of body mass index with mortality of sepsis or septic shock: an updated meta-analysis. J Intensive Care. 10.1186/s40560-023-00677-0 37400897 10.1186/s40560-023-00677-0 PMC10316562

[55] Costantini E, Carlin M, Porta M, Brizzi MF (2021) Type 2 diabetes mellitus and sepsis: state of the art, certainties and missing evidence. Acta Diabetol 58:1139 33973089 10.1007/s00592-021-01728-4 PMC8316173

[56] Oka T, Oka K, Saper CB (2003) Contrasting effects of E type prostaglandin (EP) receptor agonists on core body temperature in rats. Brain Res. 10.1016/S0006-8993(03)02268-6 12663095 10.1016/s0006-8993(03)02268-6

[57] Ueno R, Narumiya S, Ogorochi T (1982) Role of prostaglandin D2 in the hypothermia of rats caused by bacterial lipopolysaccharide. Proc Natl Acad Sci USA. 10.1073/pnas.79.19.6093 6964402 10.1073/pnas.79.19.6093 PMC347059

[58] Wang TA, Teo CF, Åkerblom M et al (2019) Thermoregulation via temperature-dependent PGD2 production in mouse preoptic area. Neuron. 10.1016/j.neuron.2019.04.035 31319050 10.1016/j.neuron.2019.06.026

[59] Fonseca MT, Rodrigues AC, Cezar LC et al (2016) Spontaneous hypothermia in human sepsis is a transient, self-limiting, and nonterminal response. J Appl Physiol. 10.1152/japplphysiol.00004.2016 26989218 10.1152/japplphysiol.00004.2016

[60] Rumbus Z, Matics R, Hegyi P et al (2017) Fever is associated with reduced, hypothermia with increased mortality in septic patients: a meta-analysis of clinical trials. PLoS ONE. 10.1371/journal.pone.0170152 28081244 10.1371/journal.pone.0170152 PMC5230786

[61] Carpenter KC, Zhou Y, Hakenjos JM et al (2020) Thermoneutral housing temperature improves survival in a murine model of polymicrobial peritonitis. Shock. 10.1097/SHK.0000000000001551 32433210 10.1097/SHK.0000000000001551 PMC7566308

[62] Lang GP, Ndongson-Dongmo B, Lajqi T et al (2020) Impact of ambient temperature on inflammation-induced encephalopathy in endotoxemic mice—role of phosphoinositide 3-kinase gamma. J Neuroinflamm. 10.1186/s12974-020-01954-7 10.1186/s12974-020-01954-7 PMC7541275 33028343

[63] Ndongson-Dongmo B, Lang GP, Mece O et al (2019) Reduced ambient temperature exacerbates SIRS-induced cardiac autonomic dysregulation and myocardial dysfunction in mice. Basic Res Cardiol. 10.1007/s00395-019-0734-1 31016449 10.1007/s00395-019-0734-1

[64] Steiner AA, Romanovsky AA (2019) Energy trade-offs in host defense: immunology meets physiology. Trends Endocrinol Metabol 30:875 10.1016/j.tem.2019.08.012 31668960

[65] do Amaral-Silva L, Gargaglioni LH, Steiner AA et al (2021) Regulated hypothermia in response to endotoxin in birds. J Physiol. 10.1113/JP281385 33823064 10.1113/JP281385

[66] Helbing DL, Stabenow LK, Bauer R (2022) Mouse sepsis models: don’t forget ambient temperature! Intensive Care Med Exp. 10.1186/s40635-022-00457-4 35773503 10.1186/s40635-022-00457-4 PMC9247123

[67] Doman M, Thy M, Dessajan J et al (2023) Temperature control in sepsis. Front Med. 10.3389/fmed.2023.1292468 10.3389/fmed.2023.1292468 PMC10644266 38020082

[68] Bradley SM, Liu W, McNally B et al (2018) Temporal trends in the use of therapeutic hypothermia for out-of-hospital cardiac arrest. JAMA Netw Open. 10.1001/jamanetworkopen.2018.4511 30646357 10.1001/jamanetworkopen.2018.4511 PMC6324404

[69] Sun YJ, Zhang ZY, Fan B, Li GY (2019) Neuroprotection by therapeutic hypothermia. Front Neurosci. 10.3389/fnins.2019.00586 31244597 10.3389/fnins.2019.00586 PMC6579927

[70] Sharma D, Farrar JD (2020) Adrenergic regulation of immune cell function and inflammation. Semin Immunopathol 42:709 33219396 10.1007/s00281-020-00829-6 PMC7678770

[71] Miller ES, Apple CG, Kannan KB et al (2019) Chronic stress induces persistent low-grade inflammation. Am J Surg. 10.1016/j.amjsurg.2019.07.006 31378316 10.1016/j.amjsurg.2019.07.006 PMC6768696

[72] Rohleder N (2014) Stimulation of systemic low-grade inflammation by psychosocial stress. Psychosom Med 76:181 24608036 10.1097/PSY.0000000000000049

